# Successful removal of a vertically impacted metallic drawing pin from the bronchus of a 12-year-old boy using a modified retrieval basket technique: A case report

**DOI:** 10.1097/MD.0000000000048001

**Published:** 2026-03-13

**Authors:** Zujing Xu, Haihua Li

**Affiliations:** a Huzhou Central Hospital, Affiliated Central Hospital of Huzhou University, Huzhou, Zhejiang, China; b Department of Pediatrics, Huzhou Central Hospital, Huzhou, Zhejiang, China.

**Keywords:** bronchoscopy, drawing pin, foreign body aspiration, pediatric airway, retrieval basket technique

## Abstract

**Rationale:**

Foreign body aspiration is most common in toddlers but can occasionally occur in adolescents. Sharp metallic foreign bodies pose a particular challenge for bronchoscopic removal because of the high risk of airway injury and the limitations of standard retrieval instruments. Complex cases may require innovative extraction techniques.

**Patient concerns:**

A 12-year-old boy presented with acute respiratory symptoms, including dyspnea, chest tightness, chest pain, cyanosis, and 1 episode of vomiting. No foreign body aspiration was reported at the initial presentation. After repeated and detailed history-taking, it was revealed that the patient had accidentally aspirated a foreign body while attending class.

**Diagnoses:**

Low-dose chest computed tomography demonstrated a hyperdense foreign body in the right lower lobe bronchus. Bronchoscopy confirmed a vertically impacted drawing pin, with its plastic head completely occluding the bronchial lumen.

**Interventions:**

A modified foreign body retrieval basket technique was applied. The basket was advanced over the sharp tip of the drawing pin and tightened around the mid–upper portion of the shaft, maintaining the tip in the center of the airway. The foreign body was then extracted by simultaneous withdrawal of the flexible bronchoscope and the basket.

**Outcomes:**

A drawing pin ~3 cm in length was successfully removed without airway injury. The patient recovered uneventfully, was discharged the next day, and remained asymptomatic during follow-up.

**Lessons:**

Vertically impacted metallic airway foreign bodies may be difficult to remove using standard bronchoscopic tools. The modified retrieval basket technique provides a safe and effective option for extracting high-risk metallic foreign bodies while avoiding thoracotomy.

## 1. Introduction

Foreign body aspiration is a common cause of respiratory distress in children, particularly in toddlers.^[1]^ However, older children and adolescents may also aspirate foreign bodies during talking, coughing, or when holding small objects in the mouth. Sharp or metallic foreign bodies, although uncommon, may cause severe airway injury and are often difficult to remove using standard bronchoscopic instruments. In deeply impacted cases, complex surgical interventions, including thoracotomy, may be required.^[2]^

Here, we report a rare case of a vertically impacted drawing pin lodged in the right lower lobe bronchus of an adolescent, which was successfully removed using a modified retrieval basket technique, thereby avoiding thoracotomy.

## 2. Case report

A previously healthy 12-year-old boy accidentally aspirated a 3-cm metallic drawing pin while holding it between his lips during class. Because he was afraid of being reprimanded, he did not initially disclose the incident. Subsequently, he developed chest tightness, chest pain, dyspnea, facial cyanosis, and 1 episode of vomiting. He was brought to the emergency department approximately 7 hours after symptom onset.

**Figure 1.**
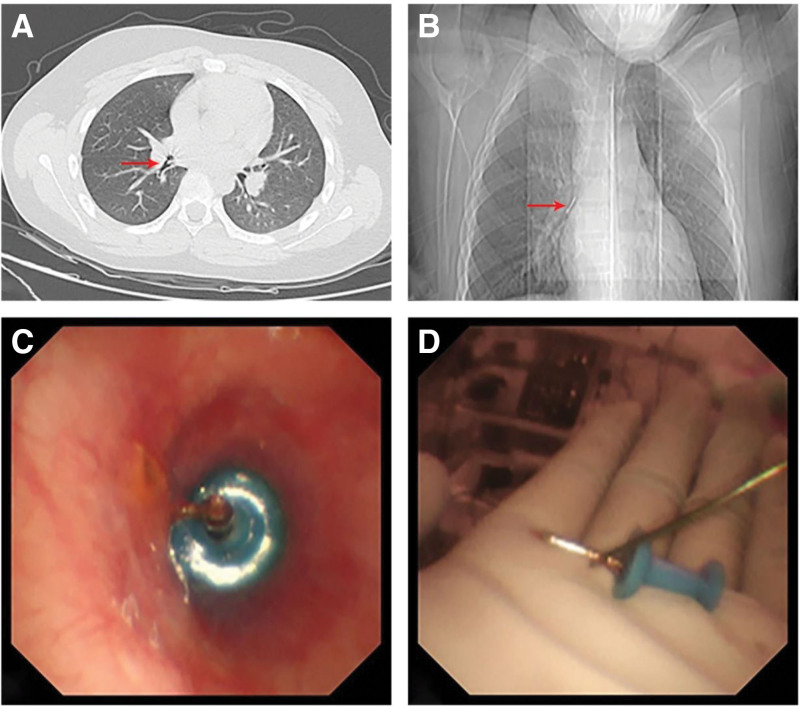
(A) CT slice showing the location of the foreign body (arrow). (B) Coronal CT reconstruction demonstrating the position of the foreign body (arrow). (C) Bronchoscopic view of a drawing pin vertically embedded in the bronchus. (D) The retrieved foreign body: a drawing pin. CT = computed tomography.

Physical examination revealed mild respiratory distress and decreased breath sounds in the right lower lung field. Vital signs were stable. Low-dose chest computed tomography was immediately performed, revealing a hyperdense foreign body in the right lower lobe bronchus (Fig. 1A, B).

Within 2 hours of presentation, rigid bronchoscopy was performed under general anesthesia. A 12-gauge rigid bronchoscope was introduced, and a flexible electronic bronchoscope (Olympus BF-P290F4.0) was advanced through the working channel. Bronchoscopic examination demonstrated a drawing pin lodged vertically within the right lower lobe bronchus, with its sharp tip pointing upward and the round plastic head downward, resulting in complete obstruction of the bronchial lumen (Fig. 1C). The shaft was slender and smooth, making secure grasping with standard forceps impossible. Multiple extraction attempts failed, and the sharp tip posed a significant risk of mucosal injury and bleeding.

To overcome this challenge, a modified foreign body retrieval basket technique was employed. The basket was introduced through the working channel of the flexible bronchoscope, opened proximal to the foreign body, and advanced obliquely along the sharp tip to the mid-portion of the shaft. The basket was then gradually tightened, allowing the wires to encircle and stabilize the mid–upper segment of the drawing pin. The position was adjusted to keep the sharp tip centered within the bronchial lumen, minimizing the risk of mucosal injury. The drawing pin, measuring approximately 3 cm in length, was safely extracted by simultaneous withdrawal of the flexible bronchoscope and the retrieval basket (Fig. 1D). No airway injury occurred.

Postoperatively, the patient’s symptoms resolved immediately. He was discharged the following day and remained free of respiratory sequelae during follow-up. During hospitalization and follow-up, the patient showed no behavioral abnormalities or psychiatric symptoms. The aspiration was considered an accidental event occurring during routine classroom activity, and no formal psychiatric consultation was deemed necessary.

## 3. Discussion

This case demonstrates the successful removal of a vertically impacted metallic drawing pin using a modified retrieval basket technique, providing a practical reference for managing complex airway foreign bodies.

### 3.1. Patient presentation and symptom characteristics

Foreign body aspiration in children typically triggers sudden and pronounced coughing and airway irritation as part of the protective airway reflex. In this case, the patient developed chest tightness, chest pain, dyspnea, and cyanosis immediately after aspiration, consistent with the typical presentation of acute airway foreign body aspiration. However, because the event occurred during class and the patient intentionally withheld the history due to fear of reprimand, the underlying cause was not immediately recognized, leading to delayed presentation.

Imaging and bronchoscopic findings revealed that the drawing pin was vertically impacted in the right lower lobe bronchus, with the plastic head completely occluding the lumen. Although this configuration may not initially result in complete airway obstruction, progressive mucosal edema and inflammatory response can rapidly worsen ventilation impairment, explaining the gradual aggravation of symptoms observed in this patient. This highlights that, even when aspiration is accompanied by typical symptoms, the specific orientation and impaction pattern of the foreign body play a critical role in disease progression.

Previous reports have described cases of sharp metallic foreign bodies, such as sewing needles and drawing pins, embedded in distal bronchi, in which standard forceps extraction failed and surgical interventions – including thoracotomy, bronchotomy, or combined bronchoscopic and surgical approaches – were required.^[3-6]^ These reports underscore the technical difficulty and high risk associated with the removal of sharp, slender, and smooth metallic foreign bodies from the bronchial tree.

Among school-aged children, holding pen caps, pen tips, or drawing pins between the lips during class is not uncommon and carries a substantial risk of accidental aspiration. Because such incidents often occur in school settings, children may fail to disclose the event promptly. Therefore, in cases of unexplained or rapidly progressive respiratory symptoms, airway foreign body aspiration should remain a key differential diagnosis, even in the absence of a clearly reported aspiration history, and early imaging should be considered.

### 3.2. Key surgical considerations

Retrieval basket techniques are increasingly used in the management of difficult pediatric airway foreign bodies, particularly when smooth or irregularly shaped objects cannot be securely grasped with standard forceps. Previous studies have demonstrated high success rates and low complication rates for basket-assisted extraction under flexible bronchoscopy.^[7]^

In this case, the technical challenges included the disk-shaped plastic head of the drawing pin, which completely occluded the bronchus and prevented the basket from passing beyond it, as well as the smooth shaft, which caused repeated slippage when grasped with forceps. In addition, the sharp tip posed a high risk of mucosal injury during manipulation.

### 3.3. Advantages of the modified retrieval basket technique

The modified retrieval basket technique offers several practical advantages in managing high-risk metallic airway foreign bodies:

1. Improved stability for smooth or sharp metallic foreign bodies

Unlike conventional forceps that rely on point-to-point grasping, the modified retrieval basket technique achieves stabilization through circumferential encasement and controlled tightening around the foreign body, rather than direct surface clamping. This approach distributes traction forces and reduces the risk of slippage associated with smooth or regularly shaped objects. In the present case, once the basket encircled the mid–upper portion of the drawing pin shaft, stable control was achieved throughout extraction, significantly improving safety and success.

2. Maintenance of coaxial traction and reduced airway injury risk

The technique helps maintain coaxial alignment among the foreign body, bronchoscope, and airway. By stabilizing the sharp tip in the center of the bronchial lumen, the risks of rotation, displacement, mucosal laceration, bleeding, and distal migration are minimized – an especially important consideration for vertically impacted, sharp metallic foreign bodies.

3. Enhanced safety of endoscopic extraction and avoidance of invasive surgery

Vertically impacted, smooth, or sharp metallic foreign bodies often require thoracotomy or airway incision when standard bronchoscopic methods fail. By enabling secure fixation and controlled coaxial traction, the modified retrieval basket technique allows safe endoscopic removal of such high-risk foreign bodies, thereby avoiding more invasive surgical interventions, reducing procedural risk, and shortening recovery time.

4. Potential for broader and cross-disciplinary application

The underlying principles of this technique are not limited to lower airway foreign bodies. It may also be applicable in otolaryngology for managing embedded or high-risk foreign bodies in the upper airway, nasal cavity, or laryngeal vestibule, particularly when objects lack graspable structures or have smooth or irregular surfaces.

### 3.4. Clinical implications and recommendations

Although foreign body aspiration is relatively uncommon in adolescents, clinical vigilance is warranted in specific scenarios. Small objects held near the mouth during class or study may be accidentally aspirated, and patients may withhold relevant history. In such cases, unexplained or progressively worsening respiratory symptoms accompanied by suggestive imaging findings should prompt consideration of airway foreign bodies.

This report does not advocate indiscriminate suspicion of foreign body aspiration in all patients with similar symptoms. Rather, it emphasizes that delayed recognition of metallic airway foreign bodies can worsen obstruction and increase procedural risk. Early bronchoscopic evaluation, when indicated, may enable timely intervention and prevent invasive surgery. When standard forceps extraction is unsuccessful or unsafe, the modified retrieval basket technique provides a safe, minimally invasive, and effective alternative. Importantly, the “minimally invasive advantage” observed in this case results from the combination of bronchoscopy and the modified basket technique, rather than bronchoscopy alone, and may also be applicable to high-risk foreign body management in otolaryngology.

## 4. Conclusion

The modified retrieval basket technique is a safe and effective method for removing vertically impacted metallic foreign bodies from the pediatric bronchus. When conventional bronchoscopic tools fail or when the foreign body poses a high risk of airway injury, this technique should be considered as an alternative to invasive surgical procedures.

## Author contributions

**Writing – original draft:** Zujing Xu.

**Writing – review & editing:** Haihua Li.
